# Age-related differences in the impact of cannabis use on the brain and cognition: a systematic review

**DOI:** 10.1007/s00406-019-00981-7

**Published:** 2019-01-24

**Authors:** Claire Gorey, Lauren Kuhns, Eleni Smaragdi, Emese Kroon, Janna Cousijn

**Affiliations:** 1 Department of Psychology, Addiction Development and Psychopathology (ADAPT) Research Center, University of Amsterdam, P.O. box 15916, 1001 NK Amsterdam, The Netherlands; 2 Dynamics of Externalizing (DEXTER) Lab, Department of Psychology, University of South Florida, Tampa, FL USA; 3 The Amsterdam Brain and Cognition Center (ABC), University of Amsterdam, Amsterdam, The Netherlands

**Keywords:** Cannabis, Development, Cognition, Brain, Adolescence, Systematic review

## Abstract

The impact of cannabis on the adolescent compared to adult brain is of interest to researchers and society alike. From a theoretical perspective, adolescence represents a period of both risk and resilience to the harms of cannabis use and cannabis use disorders. The aim of this systematic review is to provide a critical examination of the moderating role of age on the relationship between cannabis use and cognition. To this end, we reviewed human and animal studies that formally tested whether age, adolescent or adult, changes the relationship between cannabis exposure and cognitive outcomes. While the results of this review do not offer a conclusive answer on the role of age, the novel review question, along with the inclusion of both human and animal work, has allowed for the formation of new hypotheses to be addressed in future work. First, general executive functioning seems to be more impaired in adolescent frequent cannabis users compared to adult frequent cannabis users. Second, age-effects may be most prominent among very heavy and dependent users. Third, craving and inhibitory control may not decrease as much post-intoxication in adolescents compared to adults. Lastly, adolescents’ vulnerability to reduced learning following cannabis use may not persist after sustained abstinence. If these hypotheses prove correct, it could lead to important developments in policy and prevention efforts.

**Electronic supplementary material:**

The online version of this article (10.1007/s00406-019-00981-7) contains supplementary material, which is available to authorized users.

## Introduction

The risks and benefits of cannabis is an active research area due to widespread trends in legalization. Across European and North American countries, higher perceived availability of cannabis is observed to be a robust predictor of higher frequency of use [1]. This lends concern that legalization could result in higher cannabis use and more harm particularly for adolescents—an often touted ‘vulnerable’ population [2]. With growing frequencies of use, it is essential to understand the nature and scope of the risks of adolescent cannabis use. Moreover, understanding the potentially differential impact of cannabis on the adolescent compared to the adult brain could provide valuable information for prevention and policy efforts. The central aim of this systematic review is to investigate whether age influences the effect of cannabis on cognition and the brain. To address this, we reviewed studies that formally tested whether age changes the relationship between cannabis exposure and cognitive outcomes.

While public lore is that the adolescent brain is highly vulnerable to drugs, the scientific literature remains mixed—with evidence of heightened risk and of heightened resilience. Adolescence is a period characterized by a hyperactive limbic system, involved in reward, motivation, and affective learning [3, 4]. Taken together with slower, protracted development of behavioral control [5, 6], adolescents exhibit increased behavioral approach toward rewards and less behavioral withdrawal even when met with aversive consequences [4]. This is thought to facilitate the relative fast formation of maladaptive addictive behaviors during adolescence [7]. Moreover, since the adolescent brain is still developing, adolescent cannabis use may be associated with enhanced negative effects on brain structure and function [8], particularly in areas that are strongly tied with the pharmacological effects of cannabis [2]. These factors may render adolescents more vulnerable to the development of persistent substance use disorders (SUDs) and might worsen cognitive outcomes [9, 10]. Indeed, early-onset, compared to late-onset, cannabis users experience decreased executive functioning [11] and are approximately 2–5 times more likely to experience cannabis dependence or other types of drug dependence later in life [9].

On the other hand, adolescents may also exhibit heightened resilience to the effects of cannabis. Resilience can be defined as a process of overcoming the risk-related factors through the presence of protective factors. There are several unique characteristics of adolescence that could mitigate the impact of risk-related factors. As with all SUDs, most adolescent-onset cannabis use disorders (CUDs) naturally resolve over time without treatment [12]. Therefore, increased risk for developing CUDs is eventually turned into increased resilience to persistent cannabis use-related problems. High brain plasticity during adolescence might play a central role in this resilience, as evidenced by other types of adolescent-specific resilience (e.g., high rates of recovery after brain trauma) [12]. Furthermore, social factors, such as planning to attend college [13] and parental involvement in school [14], have mitigated the effects of risk-related factors on substance use outcomes in adolescence. In this sense, risk and protective factors during adolescence may function synergistically, thereby building heightened resilience to CUDs and other adverse cannabis-related outcomes in adolescents compared to adults. These competing hypotheses of risk or resilience raise the question of whether cannabis is truly more harmful for the adolescent brain.

Several reviews have addressed this question by exploring the effect of cannabis on adolescents’ neural and cognitive functioning [2, 15–22]. Focusing on mainly human work, in the past five years, there have been five qualitative [2, 15, 17, 18, 20], two systematic qualitative [16, 22], and two meta-analytic and systematic reviews [19, 21]. Although, the age windows of early versus late onset varied widely [17, 18, 22], the conclusions were similar. Specifically, earlier onset cannabis use, compared to late-onset, led to increased neurodevelopmental disruptions that resulted in functional and/or structural changes [22]. On the other hand, Blest-Hopley et al. [21] and Scott et al. [19] conducted systematic and meta-analytic reviews that investigated both adolescents and young adult cannabis users, and the results were inconsistent with these past reviews. Blest-Hopley et al. [21] showed that both adolescent and adult cannabis users exhibited deficits compared to controls but in different brain areas, suggesting age-related risks at both developmental periods [21]. Additionally, Scott et al. [19] found small but significant effect sizes for the relationship between heavy cannabis use and cognition in both adolescents and young adults. Moreover, this effect was not moderated by age group or age of onset [19].

These reviews provide valuable information regarding age-related effects of cannabis on cognition within adolescents and young adults but not between adolescents and adults. Reviews that compared early and late-onset cannabis users often included studies that only compared early and late onset within adolescence, not compared to adulthood. Similarly, Scott et al. [19] and Blest-Hopley et al. [21] included studies that only compared cannabis using adolescents against non-using adolescents or compared cannabis using adults against non-using adults. This addresses the question of whether there is a difference in brain function and cognition for cannabis users, compared to non-users, in adolescents and in adults separately (and whether the effect sizes differed). As such, a systematic review of studies that directly compared age groups is missing and the question of whether adolescents compared to adults exhibit enhanced risk or resilience to the neurocognitive effects of cannabis use remains unanswered.

In the current review, we aimed to extend previous work by examining two critical questions: does the relationship between cannabis use and cognition differ between adolescents and adults (i.e., interaction of cannabis exposure by age) and if so, do the effects vary based on cannabis use history and intoxication state? To address these questions, we systematically reviewed rodent and human studies that directly compared age groups and treated age as a moderator for the relationship between cannabis exposure and cognition. By including rodent studies, several confounds in reviews that only included human studies can be addressed. First, human work is complicated by the fact that cannabis exposure is inherently confounded with age. For example, early-onset users, compared to late-onset, may show a greater vulnerability simply because they used cannabis for a longer period. Second, human studies rarely incorporate a prolonged abstinence period and for ethical reasons, this cannot be randomly assigned. By having control over cannabis exposure, abstinence periods, and confounding variables, animal studies allow for more causal inferences into neurobiological processes. By incorporating both rodent and human studies, we are uniquely positioned to answer the question of whether there is greater risk for or resilience to cognitive decrements in adolescent cannabis users compared to adults—a novel review approach and question.

## Method

### Study inclusion criteria and search strategy

We followed the Preferred Reporting Items for Systematic Reviews and Meta-Analyses (PRISMA) guidelines for the current systematic review [23]. A MedLine, Cochrane Library, and Psyc Info search was conducted during July of 2018 with terms related to cannabis, cognition, adolescence/adulthood, and study type (see Appendix S1 for full search strategy and syntax). One author (CG) examined whether retrieved studies met or did not meet our inclusion criteria and another author (ES) conducted a random check of 1/3 of the articles. If there was a discrepancy after initial coding, four authors (CG, ES, LK, and JC) reviewed the article to reach a consensus.

**Fig. 1:**
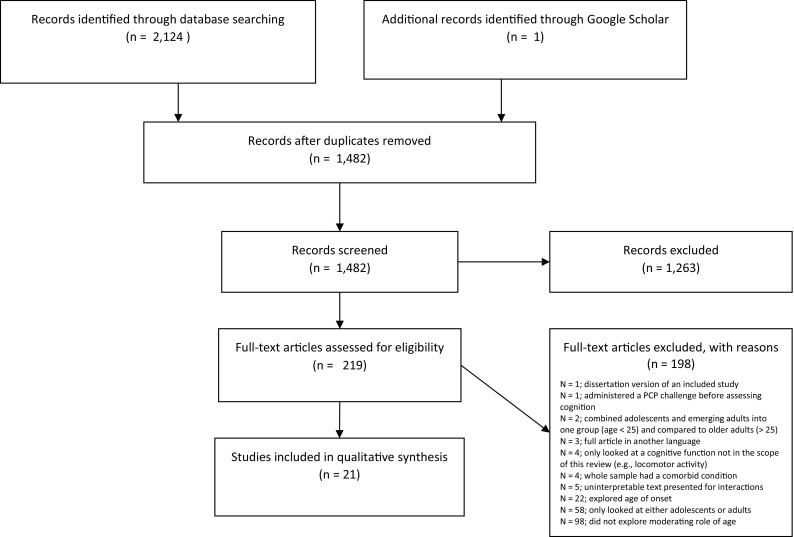
PRISMA flow diagram detailing our screening process

Inclusion criteria were: (1) human samples must have included both adolescents younger than 18 and adults older than 18, and rodent samples must have included adolescent (post-natal day 25–42 for rodents) and adult (greater than postnatal day 43 for rodents) rodents; (2) must have explored cannabis exposure as the independent variable and cognitive outcomes as the dependent variable; (3) the analyses must have included an age by cannabis exposure interaction on cognition, with age being explored either categorically (adolescent or adult) or continuously; (4) must have administered measures *during* adolescence or adulthood, not retrospectively; (5) must have used primary quantitative data collection methods (e.g., no case-studies, review papers); (6) must have solely looked at cannabis-related factors as the independent variables (e.g., did not explore cannabis-related factors in individuals with psychosis); (7) must be written in English; (8) must be published in a peer-reviewed journal before July 19th, 2018 (see Fig. 1 for a detailed screening process). Of note, we excluded studies that assessed cannabis exposure retrospectively (i.e., inclusion/exclusion criteria # 4). The studies excluded for this reason were predominantly early-onset cannabis use studies. We excluded these because age-onset variables are often inaccurate [24], with recalled age of onset increasing as an individual’s historical age increases [25]. Additionally, we decided to exclude unpublished work (i.e., inclusion/exclusion criterion # 8) to ensure that the quality of the work included in our review passed the standards of independent peer review.

**Fig. 2:**
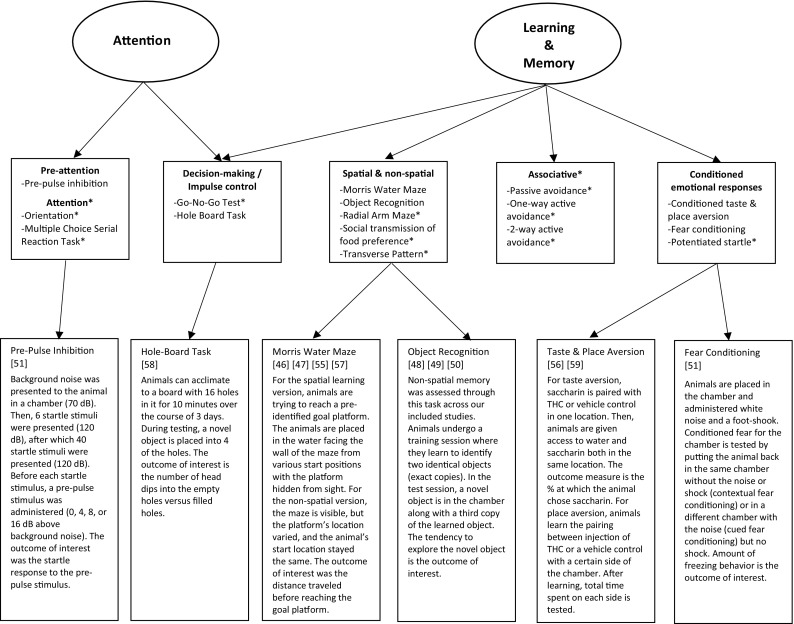
Cognitive domains assessed in rat studies across the literature are in circles, as described in a seminal review paper (see [28]). The second layer of boxes contains the narrower cognitive function along with the behavioral tasks typically used to assess that function. Asterisks represent cognitive functions or tasks that were not assessed or used in the included studies covered in this review. The third layer of boxes contains short descriptions for the tasks used across the included studies. The numbers in brackets are the citation for the study/studies that used the task. It should be noted that these are general overviews of the tasks used across our included studies; therefore, there may be slight variations in how the task was administered. Additionally, even though Pre-Pulse Inhibition is mostly used in schizophrenia research to assess sensorimotor deficits, here we mostly used and interpreted this measure as a reflection of pre-attention processes

In humans, we defined cognition as any construct that typically falls within the bounds of standard neuropsychological testing (e.g., processing speed, executive functioning). We also included more distal constructs of cognition, like craving and impulsivity, because they play a prominent role in learning, drug use, and addiction (for reviews see [26, 27]). In rodents, we defined cognition as attention, learning, and memory (see Fig. 2 for processes considered), in line with a seminal review paper [28]. However, we did not discuss findings related to the behavioral phenotype of the animal, such as locomotion and anxiety. Within the included studies, peripheral findings that did not relate to cognition were excluded from review. Additionally, reported effects irrelevant to the exploration of age as a moderator for cannabis and cognition were not included. Although, we did report post hoc testing that helped to determine the direction of the interaction.

## Results

### Study search

Our search resulted in 1482 studies once duplicates were excluded (see Fig. 1). 1263 studies were excluded after reviewing the abstracts. 198 studies were excluded after reviewing the full text. After this process, 21 rodent (*N* = 15) and human (*N* = 6) studies were included in our final qualitative review. The characteristics of the final studies are detailed in Tables 1 and 2. In addition to all standard reporting practices according to PRISMA [23], we also listed the qualitative description of the effect sizes in the studies and evaluated the quality of evidence for human work based on our own objective criteria. We included this information in Table 1.

**Table 1:** Characteristics of the human studies focused on the effect of cannabis on cognition

	Sample	Characteristics of cannabis exposure	IV(s)	DV(s)	Design	Risk, resilience, or null	Effect strength/quality
Meier et al. [29]	*N* = 1037; 52% male	Dependence diagnosis or weekly cannabis use before age 18 or after age 18	Number of dependence diagnoses throughout study (dependence @ 1 wave, @ 2 waves, or @ 3 + waves) BY age of first dependence diagnosis (before age 18 or after age 18)	Change in IQ (IQ post-cannabis initiation @ age 38 minus average IQ pre-cannabis initiation @ age 7, 9, 11, 13) 7 participants initiated before age 13, so their pre-cannabis and post-cannabis IQ was adjusted accordingly	Cannabis assessments @ ages 5, 7, 9, 11, 13, 15, 18, 21, 26, 32, 38 (Diagnostic Interview Schedule or weekly cannabis use frequency) Neuropsychological assessments @ ages 7, 9, 11, 13, 38 (Wechsler Intelligence Scale for Children-Revised or for Adults-IV)	*Null* for dependence @ 1 or @ 2 waves BY age of first diagnosis *Risk* for dependence @ 3 + waves BY age of first dependence diagnosis *Interpretation* For those diagnosed with dependence @ 3 + waves, IQ declined 0.55 SD units more among those that first met dependence before age 18 compared to those that first met dependence after age 18; results replicated when using weekly cannabis use before or after age 18 as the IV, instead of a diagnosis of dependence	Medium to large/moderate
Scott et al. [30]	*N* = 4568; 44% male	Three groups: (1) no history of use; (2) use “1–2 times per week” or less the past year; (3) “3–4 times per week” or “Daily or Almost Daily” use over the past year	Age (14–21) BY cannabis group (3 levels) BY cognitive domains (4 levels)	Cognitive function (executive control, memory, complex cognition, social cognition) assessed through the Penn Computerized Neurocognitive Battery	Participants were drawn from a sample of 50,293 adolescents and young adults who were part of the Philadelphia Neurodevelopment Cohort	*Null* for age BY cannabis group on memory, complex cognition, and social cognition *Risk* for age BY cannabis group on executive control *Interpretation* Daily or almost daily users performed worse in executive control than non-users at younger but not older ages	Small/moderate
Lee et al. [31]	*N* = 269; 55% male adults; 88% male teens	A mean of 10.7 and 22.2 on past 30-day cannabis use for adolescents and adults, respectively	Age [(165 adolescents (12–18 years old) vs. 104 adults (18 and up years old))] BY time point (intake vs. treatment) BY type of discounting (cannabis vs. money)	Delay discounting rates to cannabis and money paradigms	Participants whom were part of an outpatient treatment program completed questionnaires and delay discounting tasks before and after treatment	*Null* for age BY time point BY type of discounting *Risk* for age BY time point *Interpretation* Adolescents had smaller decreases in delay discounting for cannabis and money pre to post-treatment than adults	Not presented/weak
Albertella et al. [32]	*N* = 124; 42% male	Two groups: (1) used cannabis less than once per week or no use in the past 6 months; (2) used once a week or more often	Age (14 or 15*–24) BY cannabis group (2 levels) *Note: different age ranges 14–24 and 15–24 were mentioned in text	Negative priming (location-based negative priming task)	The study was entirely online and programs were run through Inquisit software	*Risk* for age BY cannabis use on negative priming for weekly users *Interpretation* Young and weekly users exhibited reduced negative priming compared with older and frequent users Null for age BY cannabis use on negative priming for less than weekly users	Small to medium/weak
Mokrysz et al. [39]	*N* = 40; 100% male	Medicinal cannabis—sativa strain (THC 12%) and placebo (THC < 0.3%) A mean of 10.58 and 7.94 days per month for adolescents and adults, respectively	Age [(20 male adolescents (16–17 years old) vs. 20 male adults (24–28 years old))] BY cannabis treatment (placebo vs. active)	Cognitive disorganization (VAS), alertness (VAS), craving (VAS), spatial memory accuracy and reaction time(N-Back), prose recall (subtest of Rivermead Behavioural Memory test battery), inhibitory control accuracy and reaction time (Stop Signal)	Placebo-controlled, double-blind cross-over design with vaporized placebo and vaporized active cannabis (12% THC) 24-h abstinence period before testing (not biologically verified)	*Null* for age BY cannabis treatment on spatial memory accuracy *Resilience* for age BY cannabis treatment alertness, for cognitive disorganization, for spatial memory reaction time, and for immediate and delay prose recall *Interpretation* Adolescents showed increased alertness, spatial memory reaction time, and immediate and delayed prose recall as well as decreased cognitive disorganization following intoxication compared to placebo Null for age BY cannabis treatment on inhibitory control reaction time *Risk* for age BY cannabis treatment craving and inhibitory control *Interpretation* Adolescents showed increased craving pre to post-intoxication whereas adults decreased. Adolescents exhibited reduced inhibitory control during intoxication whereas adults were unaffected	Small to medium/moderate
Padovano et al. [40]	*N* = 85; 52.8% male	Overall sample cannabis use was 21 days per month and 0.65 g per day of use	Age continuously (15–24 years old) BY report type (before cannabis, after cannabis, non-use)	Craving, alertness (VAS)	Ecological momentary assessment with several prompts per day across 14 days. The report types were categorized as: a non-use assessment; end-cannabis assessment (after cannabis use, no later than 3 h post-cannabis); begin-cannabis assessment (before cannabis use)	*Null* for age BY report type on craving *Null* for age BY report type on sedation *Resilience* for age BY report type on alertness from pre to post cannabis use *Interpretation* Age was negatively associated with change in alertness pre to post cannabis use, such that older individuals were less alert after cannabis use	Small/moderate

*IV(s)* independent variables, *DV(s)* dependent variables; *VAS* Visual Analogue Scale; *IQ* Full Scale Intellectual Quotient. Numbers in brackets correspond to the studies placement in the reference section. We determined adolescents’ risk, resilience, or null classification based on the statistical tests. The risk, resilience, or null interpretation is from the adolescent’s perspective. Only the analyses that statistically explored age as a moderator for cannabis and cognition were reported. For the DV(s) listed, the measure used to assess this cognitive domain is listed in parentheses. The effect strength was determined through standard deviation units, standardized beta-coefficients, and/or official effect sizes presented within the study’s text, with values of 0.20, 0.50, and 0.80 reflecting small, medium, and large effects, respectively. Some studies were missing this information and are, therefore, reported as “not presented”

**Table 2:** Characteristics of the animal studies focused on the effect of cannabis on the brain and cognition

	Sample	Characteristics of cannabis exposure	IV(s)		DV(s)	Design	Risk, resilience, or null
Synthetic cannabis							
O’Shea et al. [49]	*N* = 58; 100% female Wistar rats	Prolonged abstinence (21 days washout): 21 days of repeated exposure to CP or a control by injection. 0.15, 0.20, and 0.30 mg/kg for 3, 8, and 10 days, respectively	Age [(40 adolescents (30-day old) vs. 18 adults (56-day old))] BY treatment (control or CP) BY delay (2 or 6 h)	Object recognition (Novel Object Recognition Task)		Half of the rats at each developmental period were injected with the CP on each day of testing and half were tested with the vehicle	*Null* for age BY treatment BY delay on object recognition
O’Shea et al. [50]	*N* = 72; 100% male Wistar rats	Prolonged abstinence (28 day washout): 21 days of repeated exposure to CP or a control by injection. 7 days per dosage (0.15, 0.20, 0.30 mg/kg)	Age [(24 perinatal (4 days old) vs. 24 adolescents (30 days old) vs. 24 adults (56-day old))] BY treatment (control or CP) BY delay (2, 6, or 48 h)	Object recognition (Novel Object Recognition Task)		Half of the rats at each developmental period were injected with the CP on each day and half were injected with the vehicle	*Null* for age BY treatment BY delay on object recognition
Gleason et al. [51]	*N* = 40; C57BL6 mice (sex not presented)	Prolonged abstinence (110 day and 77-day washout for adolescents and adults, respectively): 3–5 day or 10-day exposure to 1 daily injection of WIN at 2 mg/kg or control	Age [(20 adolescents (30–35 days old) vs. 20 adults (63–70 days old))] BY treatment (control)	Cued and contextual fear conditioning; PPI		Half of the rats at each development time window were administered WIN; half were administered the vehicle control. After exposure, rats had a washout until they reached post-natal day 120, at which point they were administered conditioning tasks and PPI measures	*Null* for age BY treatment on contextual or cued fear conditioning after the 3- to 5-day exposure *Risk* for age BY treatment on contextual and cued fear conditioning and pre-pulse inhibition after the 10-day exposure *Interpretation* Adolescents showed reduced PPI (at pre-pulses of 4 dB and 8 dB) and contextual and cued fear conditioning
Bambico et al. [54]	*N* unclear; 100% male Sprague–Dawley rats	Prolonged abstinence (20 day washout): 20-day exposure to WIN at 0.2 mg/kg or 1.0 mg/kg or to a control	Age [(adolescents (30–50 days old) vs. adults (50–70 days old))] BY treatment (0.2 mg/kg WIN, 1.0 mg/kg WIN, or vehicle) Number of adolescent and adult animals missing	Firing rate of serotonergic activity in DR 5-HT neurons and noradrenergic activity in LC (extracellular single unit recording was used)		Adolescents and adult rats were split into 3 groups and received either 0.2 mg/kg WIN, 1.0 mg/kg WIN, or control	*Risk* for age BY treatment on serotonergic activity in the DR 5-HT neurons at 0.2 mg/kg and at 1.0 mg/kg *Interpretation* Adolescents experienced a greater reduction in serotonergic activity in the DR at 0.2 mg/kg and at 1.0 mg/kg compared to adults *Null* for age BY treatment on noradrenergic activity in the LC
Acheson et al. [57]	*N* = 22; 100% male Sprague–Dawley rats	No prolonged abstinence (no washout): 5-day exposure to either 1.0 mg/kg of WIN or control	Age [(11 adolescents (30-day old) vs. 11 adults (65-day old))] BY treatment (control or WIN55212-2) BY day (5 days) before and after controlling for anxiety-related behavior	Spatial learning (Morris Water Maze)		Half of the rats at each developmental period were injected with the WIN on each day of testing and half were tested with the vehicle For behavioral testing, animals were given 4 trials per day for 5 days	*Null* for age BY treatment BY day on spatial learning after controlling for anxiety
Fox et al. [58]	*N* = 79; 100% male Sprague–Dawley rats	No prolonged abstinence (30 min washout): a single dose of 3.0 mg/kg WIN or control 7-day exposure to one injection of 3.0 mg/kg WIN or control	Age [(46 adolescents (30-day old) vs. 33 adults (weighed 200–224 g; age not mentioned))] BY treatment (control or WIN)	Novelty seeking (Hole Board Task)		For the single dose: 12 adolescent and 6 adult rats received WIN whereas 12 adolescent and 5 adult animals received the control For the 7-day exposure: 12 adolescent and 12 adult rats received WIN whereas 10 adolescent and 10 adult rats received the control	*Null* for age BY treatment on novelty seeking for the 1-day exposure *Null* for age BY treatment on novelty seeking for the 7-day exposure
Carvalho et al. [59]	*N* = 36; 100% male Sprague–Dawley rats	No prolonged abstinence (24 h washout): 14-day exposure to 1 daily injection of either 3.0 mg/kg of WIN or control	Age [(adolescents (27–30 days old) vs. adults (55–60 day old))] BY treatment (control or WIN) Number of adolescent and adult rats was not explicitly specified and varied by analysis in the results section	Prefrontal cortical and nucleus accumbens neuronal morphology; aversion (Conditioned Place Aversion Task)		12 rats were anesthetized post-injections to conduct neuronal morphology tests For the remaining rats (*N* = 24), half of the rats at each developmental period were injected with a vehicle across behavioral sessions and half were injected with WIN	*Null* for age BY treatment on nucleus accumbens and prefrontal cortical neuronal morphology *Inconclusive* for age BY treatment on aversion, as authors did not present interaction despite it being part of the proposed analyses
Klugman et al. [60]	*N* = 92 for whole study; 100% male Wistar rats	No prolonged abstinence (1 h washout): single dose of 1.2 mg/kg of WIN	Age [(adolescents (40-day old) vs. adults (100-day old))] BY treatment (control or WIN) Amount of animals per adolescent and adult age groups was not presented. Although 17 were used for the DVs reported in the fifth column	Scaffold protein levels (i.e., Homer); NMDAR subunits: NR1 and NR2b in the mPFC and striatum		Half of the rats at each developmental period were injected with the WIN one time and half were tested with the vehicle one time Rats were sacrificed, and the ventral striatum and mPFC were dissected	*Risk* for age BY treatment on NR1 subunits and Homer protein expression in striatum and mPFC *Interpretation* WIN administration increased NR1 expression and levels and Homer protein expression in adolescents compared to adults *Inconclusive* for age BY treatment on NR2b as significance of the interaction was not presented, despite presentation of NR1 interaction
Verdurandet al [61]	*N* = 118; 100% male Wistar rats	No prolonged abstinence (24 h washout): 1-day exposure to 0.10 mg/kg HU either on day 4 or day 14, with vehicle control being administered on the previous days 4-day or 14-day exposure to HU at 0.025 mg/kg, 0.05 mg/kg, 0.10 mg/kg, or control	Age [(5 adolescents (5–7 weeks old) vs. 6 adults (10 weeks old))] BY dose (0.025 mg/kg, 0.05 mg/kg, 0.10 mg/kg, or control) BY regimen (1-day, 4 days, 14 days)	GABA_A_ receptor binding in the nucleus accumbens, amygdala, CA1 of hippocampus, cingulate cortex, caudate putamen, and dentate gyrus of hippocampus		Adolescents and adults were randomly allocated to the five treatment groups for 4-day exposure (1-day 0.10 mg/kg and 3 days control, 0.025 mg/kg for 4 days, 0.05 mg/kg for 4 days, 0.10 mg/kg for 4 days, or control for 4 days) For 14-day exposure, adolescent and adult rats were also randomly allocated to the 5-treatment groups. The 14-day exposure group was administered vehicle for 13 days before receiving the single dose, instead of 3 days	*Null* for age BY dose BY regimen on GABA_A_ density in all brain areas
Kang-Park et al. [62]	*N* = 80; 100% male Sprague–Dawley rats	No prolonged abstinence (no washout) Application of 0.1–5 µΜ WIN was applied for 10–15 min after a baseline was recorded	Age [(12 adolescents (28–35 days old) vs. 12 adults (75–110 days old))] Treatment (WIN) was not a level of the IV as they used baseline recordings to determine pre-treatment differences	EPSC; IPSC in CA1 hippocampal neurons		Brains were removed from the rats when they were under anesthesia. After incubation, a single slice of each rat brain was transferred to a chamber where recording of neurotransmission took place before and after WIN administration (10–15 min after and again 20–25 min after)	*Null* for age BY treatment on amplitude of the evoked excitatory neurotransmission *Risk* for age BY treatment on the amplitude of evoked inhibitory neurotransmission in CA1 hippocampal neurons *Interpretation* Adolescents treated with WIN experienced a greater reduction in inhibitory neurotransmission than adults
*THC*							
Cha et al. [46]	*N* = 32, 120, and 40; 100% male Sprague–Dawley rats	No prolonged abstinence (30 min washout): experiment 1: 5 mg/kg of THC or control solution was administered for 5 days Experiment 2: 2.5 mg/kg of THC, 10.0 mg/kg of THC, or control solution was administered for 5 days Prolonged abstinence (28 day washout before testing began): Experiment 3: 5 mg/kg of THC or control was administered daily for 21 days	Experiment 1: age [(16 adolescents (30–32 days old) vs. 16 adults (65–70 day old))] BY treatment (THC vs. control) Experiment 2: age [(60 adolescents (30–32 days old) vs. 60 adults (65–70 day old))] BY treatment (THC vs. control) Experiment 3: age [(20 adolescents (30–32 days old) vs. 20 adults (65–70 day old))] BY treatment (THC vs. control)	Spatial learning, non-spatial learning for all experiments (Morris Water Maze)		Experiments 1 and 2: half of the rats at each developmental period were injected with the THC on the day of testing and half were tested with the vehicle for 5 days. 30 min after injection each day behavioral testing was conducted Experiment 3: half of the rats at each developmental period were injected with THC and half were tested with the vehicle for 21 days. After a 28-day washout, behavioral testing was conducted	Experiment 1 (no prolonged abstinence): *Inconclusive* for age BY treatment. No main effects of cannabis treatment on learning in adolescents and adults Experiment 2 (no prolonged abstinence): *Risk* for age BY treatment on learning *Interpretation* THC administration inhibited both spatial and non-spatial learning in adolescent rats to a greater degree than adult rats at both dosages Experiment 3 (prolonged abstinence): *Inconclusive* for age BY treatment on spatial and non-spatial learning. No main effects of cannabis treatment on learning in adolescents and adults
Cha et al. [47]	*N* = 128 per experiment; 50% male Sprague–Dawley rats	No prolonged abstinence (30 min washout): experiment 1: 5 mg/kg of THC or control was administered daily for 5 days Experiment 3: 2.5, 5.0, or 10.0 mg/kg of THC or control solution was administered daily for 5 days Prolonged abstinence (28 day washout): experiment 2: 5 mg/kg of THC or control was administered daily for 21 days	Experiments 1 and 2: age [(64 adolescents (25 days old) vs. 64 adults (65 days old))] BY treatment (THC vs. control) experiment 3: age [(64 adolescents (25 days old) vs. 64 adults (65 days old))] BY treatment (2.5, 5.0, 10.0, or vehicle)	Experiment 1: spatial learning Experiment 2: spatial and non-spatial learning Experiment 3: spatial learning (Morris Water Maze task)		Experiments 1 and 3: half of the rats at each developmental period were injected with the THC on the day of testing and half were tested with the vehicle for 5 days. 30 min after injection each day behavioral testing was conducted Experiment 2: Same procedure as 1 and 3 but THC and control were injected daily for 21 days. After a 28-day washout, behavioral testing was conducted	Experiment 1 (no prolonged abstinence): *Risk* for age BY treatment on spatial learning *Interpretation* THC administration following no abstinence period inhibited spatial learning in adolescent rats to a greater degree than adult rats Experiment 2 (prolonged abstinence): *Inconclusive* for age BY treatment on spatial and non-spatial learning. No main effects of cannabis treatment on learning in adolescents and adults Experiment 3 (no prolonged abstinence dose–response): *Null* for age BY treatment on spatial learning
Kasten et al. [48]	*N* = 40; 100% male, B6 and D2 mice	No prolonged abstinence (72 h washout): a 10 mg/kg of THC or control solution was administered every 72 h over the course of 24 days Prolonged abstinence (4-week washout period before testing began) Rats were re-tested with behavioral paradigms	Age [(20 adolescents (27–29 days old) vs. 20 adults (68–70 days old))] BY treatment (10 mg/kg or control) B6 and D2 mice analyzed separately	Object recognition (Novel Object Recognition Task)		Half of the rats at each developmental period were injected with the THC on the day of testing and half were tested with the vehicle for a total of 6 injections (2 of which were during behavioral tests)	*Null* for age BY treatment on object recognition with no prolonged abstinence and with prolonged abstinence for B6 or D2 mice
Moore et al. [55]	*N* = 40; 100% male; Sprague–Dawley rats	No prolonged abstinence (30 min washout): a 10 mg/kg of THC or control solution was administered daily for 5 days	Age [(20 adolescents (30–35 days old) vs. 20 adults (70–75 days old))] BY pre-treatment (10 mg/kg or control) BY challenge	Spatial learning (Morris Water Maze), CB1 hippocampal distribution (immunofluorescence), CB1 hippocampal number, CB1 coupling to downstream G protein, CB1 desensitization		Half of the rats at each developmental period were injected with the THC and half with the vehicle for 5 days. On days 6 and 10, the THC pre-treated rats were injected with another dose of THC, whereas the other rats were exposed to the vehicle again. 30 min after the injection of THC or vehicle behavioral testing was administered. For neural-related DVs, the rats were euthanized post-behavioral testing to conduct analyses	*Risk* for age BY pre-treatment BY challenge on spatial learning *Interpretation*: Adults pre-treated with THC showed lower reductions in spatial learning after the challenge then treatment-matched adolescents *Inconclusive* for age BY pre-treatment BY challenge on hippocampal CB1 number, distribution, or coupling
Schramm-Saptya et al. [56]	*N* = 64–66; 100% male, Sprague–Dawley rats	No prolonged abstinence (0–10 min washout): 0.5 or 5 mg/kg of THC or control solution was administered daily for 5 days	Age [(32–33 adolescents (28 days old) vs. 32–33 adults (64–66 days old))] BY treatment (0.5, 5 mg/kg, or control)	Aversion (Conditioned Taste Place Aversion tasks)		Half of the rats at each developmental period were injected with the THC on the day of testing and half were tested with the vehicle for 5 days. For Conditioned Taste Aversion Testing and Place Aversion Testing, rats were injected immediately after the saccharin drinking session and 10 min before being placed in the chamber, respectively	*Risk* for age BY treatment on place aversion *Interpretation* 5 mg/kg THC administration caused a greater place aversion in adolescents and adults compared to vehicle administration. However, adults spent less time in the drug-associated place than adolescents (i.e., greater place aversion). There were no age-related differences for the lower dosage *Null* for age BY treatment on taste aversion

*IV(s)* independent variables, *DV(s)* dependent variables, *WIN* WIN 55212-2, *CP* 55,940, *HU* HU210, *CB1 and CB2* cannabinoid receptor type 1 and 2, *DR* Dorsal raphe, *LC* locus coeruleus, *mGluR5* metabotropic glutamate receptors type 5, *mPFC* medial prefrontal cortex, *PPI* pre-pulse inhibition, *EPSC* excitatory postsynaptic current, *IPSC* inhibitory postsynaptic current, *CA1* cornu ammonis. Numbers in brackets correspond to the studies placement in the reference section. We determined adolescents’ risk, resilience, or null classification based on the statistical tests. The risk, resilience, or null interpretation is from the adolescent’s perspective. Only the analyses that statistically explored age as a moderator for cannabis and cognition were reported. For the DV(s) listed, the measure used to assess this cognitive domain is listed in parentheses

All authors independently reviewed and rated the quality of human evidence: (1) strong level of causality: longitudinal studies with a comparison of adolescent and adult values and that included relevant covariates; (2) moderate level of causality: studies that were longitudinal with a comparison of adolescent and adult values without accounting for relevant covariates or cross-sectional human studies with matched adult and adolescent groups that considered relevant covariates; (3) weak level of causality: studies that were cross-sectional but did not have matched adolescent and adult groups and/or did not consider relevant covariates. Notably, we did not rate the quality of rodent work. Given the enhanced experimental control, the quality was very similar (and strong) across rodent studies. In the discussion, we did provide an overview of the limitations that reduce the generalizability of the rodent work overall.

### Human studies on cannabis and cognition

Human studies explored the role of age on the relationship between some form of cannabis use history (e.g., dependence/amount past use) and cognition or alternatively, on the relationship between cannabis intoxication and cognition. These two types of studies are discussed separately below.

#### Age, history of repeated cannabis exposure, and cognition

Four human studies assessing the relationship between history of repeated cannabis exposure and cognition met our inclusion criteria (see Table 1 for study characteristics). Meier et al. [29] investigated participants over the course of 33 years and were interested in the persistence of cannabis dependence (total number of times a cannabis dependence diagnosis was met across 5 study waves; 1, 2, or 3 +) by age of first diagnosis (before age 18 and after age 18) on change in intelligence quotient (IQ). Past year cannabis dependence was assessed through a diagnostic interview at age 18, 21, 26, 32 and 38, and IQ was assessed before and after the initiation of cannabis use at age 7, 9, 11, 13 and 38 (Wechsler Intelligence Scale for Children-Revised or for Adults-IV). There were no effects of age of first diagnosis on change in IQ (i.e., post-cannabis IQ minus average IQ before cannabis initiation) with only 1 or 2 diagnoses of cannabis dependence. However, individuals that had a dependence diagnosis at 3 or more waves and that met their first diagnosis before age 18 experienced a 0.55 standard deviation reduction in IQ compared to those that had a first diagnosis after age 18. Meier et al. [29] repeated this analysis using weekly cannabis use (instead of diagnosis of dependence) before or after age 18 on change in IQ and results were similar [29].

Like Meier et al. [29], three separate studies found age-related cognitive vulnerabilities in adolescents but only for heavier cannabis users. More specifically, Scott et al. [30] administered the Penn Computerized Neurocognitive Battery to almost daily and daily cannabis users (~ 3–7 times per week) and discovered that adolescents (i.e., ages 14–17) compared to adults (i.e., ages 18–21) exhibited lower executive control, consisting of sustained attention and working memory subtests, in comparison to the non-user control group. However, this age-related deficit did not extend to weekly cannabis users (1–2 times per week or less) when compared to the non-user group. Additionally, there was no age by cannabis effect on other cognitive domains, including memory (i.e., verbal episodic, face, and spatial episodic memory), complex cognition (i.e., mental flexibility, language reasoning, nonverbal reasoning, and visuospatial ability), or social cognition (i.e., emotion identification, emotion differentiation, and age differentiation). However, weekly cannabis users, but not almost daily or daily users, performed better than non-users in executive control, memory, and social cognition, suggesting a potential positive effect for weekly cannabis users regardless of age [30].

Lee et al. [31] discovered that adolescents, compared to adults, in outpatient therapy for CUDs showed a smaller reduction in bias toward immediate, smaller cannabis and monetary rewards from pre to post treatment, as measured by a delay discounting task. Adolescents and adults both showed decreased reductions in bias towards cannabis compared to money from pre to post treatment, suggesting no age-related differences in delay discounting based on the type of reward presented. These results suggest that adolescents with CUDs, compared to adults, exhibit less positive change in impulsive responding to cannabis and money from pre to post-treatment [31]. Lastly, although not one of their central questions, Albertella et al. [32] cross-sectional study analyzed the effect of continuous age (15–24 years old) by cannabis use frequency, defined as less than once a week or more than once a week over the past 6 months, on the ability to detect relevant targets amongst distracting stimuli in a location-based negative priming task. There was no main effect of age on negative priming. However, there was an interaction between age and cannabis use frequency such that weekly younger users showed lower negative priming scores, or lower accuracy in detecting relevant targets amongst distracting stimuli, compared to weekly older users. This effect did not extend to monthly users, suggesting compromised inhibitory control in younger, weekly users specifically [32].

We categorized these four studies as only meeting a weak-to-moderate level of causality. Additionally, we assessed the strength of the significant age by cannabis interactions with the reported standardized beta coefficients or z-scores. The effects were small (0.20 or less) for Scott et al. [30], small to medium (between 0.20 and 0.50) for Albertella et al. [32], and medium to large (between 0.50 and 0.80) for Meier et al. [29].

Meier et al. [29] study was classified as a moderate level of causality. The analyses were between-subject, and thus, it could be that other variables not accounted for are associated with adolescent-onset cannabis dependence and the time-varying effect of IQ [29]. For instance, two commentaries concluded that the effect could be accounted for by a simulation model of the confound, socioeconomic status [33], and by personality traits [34]. These arguments were later rebutted by a commentary from the original authors, who showed that effects replicated when only looking at subjects in the middle socioeconomic class and after accounting for self-control [35]. However, since the age-related analyses are based on a between subject factor, the study was not classified as having a strong level of causality. Scott et al. [30] study was classified as a moderate level of causality as well. Their effects were robust beyond numerous confounds, including socioeconomic status. However, given the cross-sectional nature, it is impossible to determine if the observed deficits were pre-existing [30].

Lee et al. [31] study was classified as weak level of causality. Adolescents showed a smaller decrease in bias toward immediate cannabis-related and monetary rewards from pre to post treatment. These results could indicate poorer cognitive recovery in adolescents. If true, it is surprising given that adults used twice the amount of cannabis than adolescents in their sample (22.2 days for adults vs. 10.7 days for adolescents; [31]). Alternatively, though, it may mean that adolescents are resistant and unmotivated to therapy in general. Adolescents are rarely self-referred to treatment for CUDs. Moreover, it has been shown that motivation, pre-treatment expectations of positive change, and therapist–client relationship account for most of the variance in client outcomes, with actual treatment modality only accounting for 1% of the variance in symptom reduction [36, 37]. Additionally, it could also be that adolescents may have reached a developmental ceiling for delay discounting with treatment, and therefore, could not reach the same level of reduction in delay discounting as adults. This is in line with literature suggesting that adolescent controls, compared to adult controls, displayed higher rates of delay discounting to money [38]. Without control groups for adolescents and adults within their investigation, it is impossible to determine how age-matched peers without CUDs would perform in delay discounting tasks. Along with these issues of interpretation, we also classified the quality of the evidence for causality as weak due to several methodological issues. Specifically, the unmatched groups regarding past cannabis use (22.2 days of cannabis use per month for adults, 10.7 for adolescents), motivation or expectancy for therapy, gender (88% male for adolescent, 55% male for adults), and group size (*N* = 165 for adolescents, *N* = 104 for adults), as well as the use of different treatment modalities across groups all limited the study’s evidence of causality. Similarly, Albertella et al. [32] study was classified as weak evidence for causality because (sub)acute effects of cannabis intoxication might have confounded the findings as 37% of the weekly and 3% of the less than weekly users used cannabis within the past 24 h.

Overall, these four studies suggest that adolescent weekly to daily cannabis users experience greater reductions in general executive functions like working-memory and attention, which may in turn affect cognitive tasks that rely on these functions. The effect in one study seems to extend to IQ in individuals with persistent cannabis dependence (diagnosis at 3 or more study waves) with a first diagnosis before age 18. This further supports the hypothesis that age-related deficits in executive functioning and Full-Scale IQ are probably most noticeable in the most heavy and problematic cannabis users. However, it is unclear if these age-related deficits would extend beyond a prolonged abstinence period. Specifically, none of these studies had a standardized abstinence period. Therefore, intoxication levels during the cognitive assessment might vary between participants within studies as well as between studies. For the studies of Scott et al. [30] and Lee et al. [31], it is unclear whether (sub)acute effects of intoxication could have affected the results, as no measure of recent cannabis use was included. Meier et al. [29] and Albertella et al. [32] did assess past 24-h cannabis use and took this into account in part of their analyses. Meier et al. [29] showed that their main results did not change significantly after exclusion of past 24-h cannabis users. Albertella et al. [32] showed that across all participants, past 24-h cannabis use was not a significant predictor of their main outcomes measure, negative priming, but did not take into account the existing group difference in past 24 h use. Given this confound, the limited amount of studies, and the strength and quality of evidence, results should be considered preliminary.

#### Age, cannabis intoxication, and cognition

Two human studies, that met our inclusion criteria (see Table 1 for study characteristics), conducted studies on the effects of cannabis intoxication in current adolescent and adult cannabis users. Notably, only one of these studies [39] had a standardized abstinence period, although it was only 24 h and was not biologically verified. Mokrysz et al. [39] administered both vaporized cannabis (12% THC) and a placebo control, in separate sessions, to a matched sample of 20 male adolescents (16–17 years old; mean of 10.58 days of cannabis use per month) versus 20 male adults (24–28 years old; mean of 7.94 days of cannabis use per month). Interestingly, adolescents showed signs of both risk and resilience. Adolescents showed less memory impairments, both immediate and delayed. That is, adults showed twice as large of a reduction in delayed prose recall following intoxication, compared to placebo, as well as lower immediate prose recall than adolescents. Moreover, adolescents did not show a difference in reaction time to a spatial N-back working memory task during intoxication compared to placebo; whereas, adults were significantly slower during cannabis intoxication compared to placebo. There were no age-related differences for spatial N-Back accuracy. Lastly, adults reported significantly higher cognitive disorganization and significantly lower alertness on a visual analogue scale while intoxicated than adolescents.

In contrast to the working memory findings, adolescents showed heightened risk for craving (measured by a visual analogue scale) and accuracy, but not reaction time, in an inhibitory control task (measured by a stop signal task). Specifically, pre- to post-intoxication craving increased in adolescents but decreased in adults. Moreover, adults’ inhibitory control score was unaffected by cannabis; whereas, adolescents showed reduced inhibitory control accuracy when intoxicated [39]. In a partial replication of Mokrysz et al. [39], Padovano et al. [40] conducted an Ecological Momentary Assessment in cannabis users, aged 15–24 years old, who averaged roughly 21 days of cannabis use per month and 0.65 g per use day. The researchers tracked participants across 14 days and administered craving and alertness measures via a wireless device that delivered several prompts each day. Researchers then categorized their assessments as during non-use days, before cannabis use, or after cannabis use [40]. In line with Mokrysz et al. [39] age was negatively associated with change in alertness post-cannabis use, such that younger individuals were more alert relative to older individuals. This effect did not extend to sedation. Unlike Mokrysz et al. [39], there was no effect of age on craving from pre- to post-cannabis use.

We rated both studies as moderate evidence of causality, given the sound experimental design and control of confounding variables. The strength of the effects for Padovano et al. [40] were small (less than 0.20), as determined by the presented standardized beta coefficients. For Mokrysz et al. [39], the strength of the effects were medium (0.20–0.50) for alertness, immediate/delay prose recall, reaction time to the N-back task, and inhibitory control accuracy and were small (less than 0.20) for craving, as determined by the presented eta-squared values. Given Mokrysz et al. [39] finding of reduced cognitive impairment post-intoxication in adolescents, one could hypothesize that this would be beneficial to negative outcomes of acute intoxication (e.g., risky driving) as well as long-term cognition. Therefore, this resilience effect of cannabis on adolescents’ cognition when intoxicated contradicts the risk effects observed in non-intoxicated cannabis users. However, Mokrysz et al. [39] finding for inhibitory control is consistent with the studies reviewed above. This suggests that age effects may vary based on intoxication state and discrepancies may only appear for certain cognitive functions.

In addition, the observed effects of age on craving conflict; however, this could be due to methodological differences across the two studies. Specifically, Mokrysz et al. [39] treated age dichotomously (adolescents vs. adults), whereas Padovano et al. [40] treated age continuously. Furthermore, the cannabis users in Padovano et al. [40] sample exhibited two times higher frequency of cannabis use than Mokrysz et al. [39] sample. These methodological influences may have had more of an impact in their discrepant findings particularly because the effect for craving was small in Mokrysz et al. [39] study.

Overall, the results of these two studies of current cannabis users under acute cannabis intoxication appear to contradict each other for craving but not alertness. Additionally, except for the inhibitory control outcome, Mokrysz et al. [39] findings on cognition differed from included human studies that did not incorporate acute intoxication. It is possible that age intersects with acute intoxication differently, thereby leading to distinct patterns with cognitive outcomes compared to non-acute intoxication investigations.

### Animal studies on cannabis and cognition

Across animal work, researchers typically expose rodents to daily cannabinoid injections, including tetrahydrocannabinol (THC), the main compound in natural cannabis, or synthetic cannabis. The reviewed THC administration studies are most relevant to the human studies described above, of which none considered the use of synthetic cannabinoids. Findings for synthetic cannabinoid administration studies do not necessarily directly translate to the effects of natural and synthetic cannabinoids in humans. However, such studies are important because they provide insight into synthetic cannabinoids, which are studied very minimally in human work. Animal studies that tested cognition in repeatedly exposed rodents during THC intoxication are most comparable to human studies that tested cognition post-cannabis intoxication (see [39, 40]). Rodent studies that tested cognition in repeatedly exposed rodents after prolonged abstinence are most comparable to human studies that tested abstinent cannabis users [31]. In terms of the translation of rodents to human development, rats and mice have similar developmental periods [41] and are considered ~ 10–18-years-old during post-natal day 25–42 (i.e., adolescence) and 18–25-year-old during post-natal day 43–65 (i.e., emerging adulthood; [42]). Given the fast development, it is common for researchers to only administer THC or synthetic cannabis for 5 days, which is roughly comparable to 6 months of daily exposure in humans [43].

Researchers studying rodents use a variety of measures designed to match human cognitive functions. In Fig. 2, we present a more in-depth description of the tasks used across the included rodent studies and what domain of cognition they target. Fifteen rodent studies met our inclusion criteria (see Table 2 for study characteristics), of which five administered THC and ten administered a synthetic cannabinoid. Across studies, half of the rodents at each development period were administered the active cannabinoid and half were administered a vehicle control. Synthetic cannabinoid studies used compounds such as CP 55,940 (CP), WIN 55212-2 (WIN), and HU210 (HU). All these compounds interact with both Cannabinoid (CB) 1 and CB2 receptors, like the main active ingredient in natural cannabis (i.e., THC), but are significantly more potent [44, 45]. All included animal studies used rodents and neurocognitive assessments were performed during acute intoxication or after prolonged abstinence. The results of these studies are discussed separately below.

#### Age, repeated cannabinoid exposure, and cognition after prolonged abstinence

Three experiments explored the effect of repeated THC exposure on learning and/or memory after prolonged abstinence. To investigate the effects on spatial and non-spatial learning through the Morris Water Maze, Cha et al. [46] administered 2.5 mg/kg THC or 10.0 mg/kg THC to male Sprague Dawley rats for 21 days (1 injection per day) [46] and then, extended their design to a mixed-gender sample [47]. For both of their experiments, the authors did not provide statistics for their age by cannabis treatment interaction, despite proposing it in their statistical analyses. However, there were no main effects of cannabis treatment (THC vs. control) on spatial and non-spatial learning in adolescents or adults across both studies [46, 47]. Kasten et al. [48] administered 10 mg/kg THC or control vehicle to male B6 and D2 mice across 24 days (1 injection every 72 h) and found no evidence of age-related differences on object recognition, as measured by the Novel Object Recognition test, following repeated THC administration and a 4-week washout period. No overall main effect of treatment emerged across the age groups [48].

Four experiments investigated age-related differences of the effect of synthetic cannabinoid exposure after prolonged abstinence. To investigate working memory, O’Shea et al. [49] administered different dosages of CP to female Wistar rats for 21 days (1 injection per day). After 21 days of abstinence, there was no age by cannabis treatment effect or main effect of treatment on working memory across age groups, as measured by the Novel Object Recognition Task [49]. In a follow-up study, O’Shea et al. [50] repeated the same protocol in male Wistar rats with a 28-day abstinence period. CP reduced working memory in both adolescents and adult CP-treated rats compared to vehicle, but like their study in females, age did not moderate the effect of CP on working memory [50].

Gleason et al. [51] investigated the effect of WIN administration (3- to 5-day exposure and 10-day exposure) on cued and contextual fear conditioning and sensorimotor gating in adult and adolescent C57BL6 mice. After prolonged abstinence, there was an age by cannabis treatment effect in the 10-day exposure group only. Specifically, adolescent mice showed increased reductions in sensorimotor gating (a measure of attentional abnormalities) and cued and contextual fear-conditioning, while no impairments were found in adult mice [51]. Lastly, Bambico et al. [54] administered WIN to male Sprague–Dawley for 20 days to investigate its effects on the firing rates of serotonergic and noradrenergic activity in dorsal raphe neurons and locus coeruleus neurons (areas involved in appetitive and aversive information processing; see [52, 53]). After a 20-day washout, they observed a moderating effect of age on serotonergic activity in the dorsal raphe but not on noradrenergic activity in the locus coeruleus. Specifically, adolescents experienced a significantly greater reduction in serotonergic activity in the dorsal raphe compared to adults. While there was no significant interaction for noradrenergic activity, main effects suggested that adolescents, not adults, experienced significant enhancement in noradrenergic firing rates in a dose-dependent manner following cannabis exposure [54].

Following prolonged abstinence, the THC and synthetic studies suggest that age does not moderate the effects of THC on memory. Additionally, for spatial and non-spatial learning, THC, relative to control, does not affect spatial learning or non-spatial learning in adolescent and adults after prolonged abstinence. It is unclear if age changes this relationship, as interaction effects were inconclusive. There appeared to be a different pattern for neural and behavioral outcomes more closely linked with emotional processes (i.e., fear conditioning and appetitive and aversive information processing), with adolescents showing increased neural and behavioral reductions if exposed to synthetic cannabis. It is unclear if this extends to natural THC, after prolonged abstinence, as this was not explored.

### Age, repeated cannabinoid exposure, and cognition during intoxication

Seven experiments explored the effect of 5-day THC exposure without a prolonged abstinence (minimal or no wash-out) period on learning and/or memory. Behavioral testing occurred 30 min after each injection, except for Moore et al. [55] who exposed rats to THC for 5 days, and then, administered behavioral testing on days 6 and day 10, 30 minutes after each THC injection.

For spatial and non-spatial learning (Morris Water Maze), Cha et al. [46] administered 2.5 mg/kg THC, 10.0 mg/kg THC, or control vehicle to male Sprague Dawley rats in one experiment (experiment 1) and then administered 5.0 mg/kg in another experiment (experiment 2) with a different sample of male Sprague Dawley rats [46]. In experiment 1, adolescents showed a greater reduction in spatial and non-spatial learning than adults at both dosages, despite no significant differences between adult and adolescent rats who received vehicle injection. For experiment 2, results for the interaction were inconclusive as statistical results were not presented. Cha et al. [47] extended their design in a mixed-gender sample of Sprague Dawley rats across two experiments; in experiment 1, they administered only one dosage of THC and in experiment 3, they administered three dosages of THC [47]. In experiment 1, there was a significant moderation of cannabis treatment by age with adolescent rats showing reduced spatial learning compared to adults. However, there was no age by dosage (2.5 mg/kg, 5.0 mg/kg, and 10 mg/kg) interaction on spatial learning in the third experiment, suggesting that effects of age on spatial learning are insensitive to THC potency.

In male Sprague Dawley rats, Moore et al. [55] observed that adults pre-treated with THC (5-day THC exposure) showed decreased reductions in spatial learning (Morris Water Maze) on day 6 and 10 after acute intoxication than treatment-matched adolescents. It remains inconclusive if the effects extend to neural mechanisms (e.g., CB1 hippocampal distribution) underlying this behavioral deficit. The interaction was a proposed analysis in their statistical section, but the result was not presented [55]. Nonetheless, this suggests that the age effects of 5-day THC exposure on learning remain evident up to 10 days.

Schramm-Sapyta et al. [56] were interested in whether there are age-related differences in another type of learning—aversive responses to low and high potency of THC. Notably, THC was not paired with an aversive stimulus, but rather was investigated alone as it is thought to have aversive effects at high dosages (5 mg/kg). The researchers administered 0.5 mg/kg, 5 mg/kg, or control vehicle for 5 days to male Sprague Dawley rats. There was a moderating effect of age on conditioned place aversion (cannabis treatment or vehicle control treatment paired with specific locations). Specifically, adolescent and adults both showed greater place aversion after 5 mg/kg THC administration, relative to control rats, but THC-treated adults spent less time in the drug-associated place than THC-treated adolescents, indicating greater place aversion in adults. There were no age-related differences for the lower dosage. Both age groups showed an increase in taste aversion when treated with THC at both dosages following saccharin, relative to vehicle, but there was a null effect for age by treatment on taste aversion [56]. Additionally, in a separate study of B6 and D2 male mice, there was no main effect of THC treatment on object recognition, measured by the Novel Object Recognition task, across both age groups, and there was a null effect for age by treatment on object recognition [48].

Overall, adolescents appear to be more vulnerable to learning impairment but not memory decline following THC exposure with minimal to no abstinence period. Additionally, it may be that adolescents experience reduced aversive responses to THC, thereby reducing the conditioning effects to neutral cues (i.e., place; as shown in Schramm-Sapyta et al. [56] work). This would indicate that repeated exposure to THC without an abstinence period decreases general learning and increases drug-specific learning for cannabis’ positive effects in adolescents at high dosages, compared to adults.

Six studies explored the effect of synthetic cannabinoid exposure without prolonged abstinence, two of which focused on behavior and four of which focused on neuronal changes. Acheson et al. [57] investigated the effect of WIN exposure on spatial memory in adolescent and adult male Sprague Dawley rats. WIN did not affect spatial memory and there was no age by cannabis interaction effect. Fox et al. [58] investigated novelty seeking in male Sprague–Dawley rats 30 min after WIN exposure. There was no main effect of treatment or an interaction of treatment by age on novelty-seeking behavior, regardless of exposure length (1 or 7 days; [58]). The results of these two studies suggest that there is no behaviorally measurable impact of acute WIN intoxication on spatial memory and novelty-seeking regardless of age.

To connect behavioral differences to brain changes, Carvalho et al. [59] exposed male Sprague Dawley rats to WIN for 14 days with a 24-h abstinence period and then measured conditioned place aversion (a behavioral measurement of aversive learning) and neuronal morphology (the shape and structure of neuronal components). Changes in the shape of dendrites were evaluated in the nucleus accumbens and the prefrontal cortex. Only cannabinoid exposed adult rat brains demonstrated increased dendritic length in the medial prefrontal cortex. However, age did not significantly moderate the treatment effect, indicating changes at the neuronal level did not differ between the adult and the adolescent brain. No conclusions could be drawn for any discrepancies in aversion, as authors did not present the results of the interaction analysis [59].

Three studies investigated the effects of synthetic cannabinoids on neuronal activity in the brains of rats after acute intoxication with mixed results. Klugman et al. [60] measured changes in the level of NMDA receptors (subunits NR1 and NR2b) and Homer protein levels in male Wistar rats. Both are involved in synaptic plasticity and are important for processes such as learning and memory at the cellular level. In both the striatum and medial prefrontal cortex of WIN treated rats, they found a differential effect of age on treatment with an increase in the NR1 subunit in adolescents but a decrease in adults. They also observed a slight reduction in levels of the NR2 subunit in the medial prefrontal cortex, but they did not report the result of the age by treatment interaction. Homer protein levels were also significantly more elevated in adolescents compared to adults [60]. Verdurand et al. [61] investigated the effect of synthetic cannabinoid HU210 on GABA_A_ receptor density—the chief inhibitory compound of the brain—after exposure for 1, 4, or 14 days. They observed higher density of GABA_A_ receptors in the brains of 4-day treated rats than the 14-day treated rats. However, there was no differential impact of age group [61]. Kang-Park et al. [62] applied WIN to brain slices of Sprague Dawley rats and measured inhibitory and excitatory activity of neurons in the hippocampal CA1 region. Although acute intoxication with WIN significantly decreased the excitatory activity in both adolescent and adult rats, there was not a differential effect on adults and adolescents. However, WIN-treated adolescents experienced a greater reduction in inhibitory activity than adults. Reduced inhibition of inhibitory neurotransmission can be understood as increased sensitivity to exogenous cannabinoid-mediated effects at the neuronal level [62]. Overall, all three studies found some evidence for a differential impact of synthetic cannabinoids on neuronal activity based on age.

## Discussion

Our systematic review of human and animal studies specifically aimed to address whether age moderates the relationship between cannabis use and cognition. This question is of particular importance given the scientific debate around whether adolescents, compared to adults, are at heightened risk or are resilient to potential harms of cannabis use and dependence. Integrating both human and animal work, we found preliminary evidence for an age-dependent effect of cannabis that varied based on cannabis use history and intoxication state. However, given the paucity of studies, multiple research gaps exist that need to be addressed in future studies before any strong conclusions can be made.

Interestingly, the human and rodent studies investigating the direct effects of cannabinoid intoxication on cognition showed preliminary evidence for both risk and resilience during adolescence. Human adolescents exhibited less impairment in memory post-intoxication than adults. On the other hand, they also showed greater impaired inhibition and interestingly, less of a craving reduction after intoxication than adults [39]. Similarly, rodent adolescents exhibited increased drug-specific learning for the positive effects of cannabis compared to adults [56]. Combined with reductions in inhibitory control, adolescents’ higher craving after cannabis intoxication may promote binge-like behavior, rending adolescents more susceptible to using larger quantities of cannabis over a short period of time than adults. This is in line with research indicating increased prevalence of binge drinking amongst adolescents compared to adults [63] and may be partly driven by adolescents’ increased responding to the appetitive vs. aversive effects of drugs [4]. Nevertheless, these findings are extremely tentative given that this is based on one human study. Multiple replication studies are needed before any firm hypotheses can be drawn regarding age-related differences in craving post-cannabis use.

Regarding effects of long-term cannabis use, we found limited evidence that repeated cannabis use is associated with larger impairments in executive functioning and IQ in adolescents compared to adults. It is important to note that this effect may only emerge when comparing almost daily using adolescents and adults. These results align with previous reviews which suggested that early vs. late-onset cannabis use led to increased neurodevelopmental and cognitive disruptions [17, 18, 22]. However, they partially contradict a recent meta-analysis by Scott et al. [19], which found small but comparable decreases in neuropsychological domains in both adolescent and young adult cannabis users overall. Scott et al. [19] compared effect sizes of separate adult and adolescent studies, while we focused solely on studies that made direct comparisons between adolescent and adult cannabis users. This approach is necessary to draw conclusions about whether adolescents are at *heightened* risk or resilience to the potential effects of cannabis on cognition compared to adults.

Nevertheless, given the weak to moderate level of human evidence, it is too early to draw strong conclusions, especially when the findings of the reviewed animal studies are considered. In line with our human results, adolescent rodents exhibited greater deficits in learning after repeated THC exposure [46, 47, 56]; however, this effect did not emerge in studies that incorporated a prolonged abstinence period [46–50]. This suggests that the effects may be the result of (sub)acute effects of cannabis intoxication and thus, is in line with Scott et al. [19] findings that no impairments emerged in neuropsychological functioning in both adolescents and young adults when studies included a prolonged abstinence period. Moreover, it remains to be tested if the findings reflect direct effects of cannabis on functioning or whether secondary environmental effects play a role. For instance, heavy cannabis use or CUDs may disrupt the ability to pay attention in school, thereby reducing receptiveness to education. This may reflect a malleable reduction in cognition more than a long-lasting neural effect. To unravel the underlying mechanisms, extended longitudinal and genetically informed studies targeting various types of cannabis exposure (daily/almost daily; weekly; monthly; past user; never used) as well as age group comparisons (adolescents, adults) are needed.

The current review also included animal studies that investigated the effect of synthetic cannabinoids. Studies investigating behavioral effects found no differential effect of synthetic cannabinoids between adolescents and adults [57, 58]. However, studies investigating neural effects found differences between adolescents and adult rodents in protein expression and neurotransmitter activity after acute intoxication [61, 63]. These age-related differences should be interpreted cautiously as it is not possible to infer whether they are evidence of impairment or improvement and whether these findings generalize to other cannabinoids and translate to humans. Synthetic cannabinoids include a broad class of CB1 and CB2 receptor agonists and can be more potent than natural cannabinoids. A recent review suggested that its use is increasing, as is the severity of the reported adverse reactions (e.g., hospital visits and symptoms of paranoia) [64]. Unfortunately, human research into the effects of synthetic cannabinoids on cognition is still missing.

In addition to the study of the effects of synthetic cannabinoids in humans, this review highlights multiple research gaps that need to be addressed. First, none of the studies explored the effect of varying ratios of THC to cannabidiol. This is a salient gap as age-related differences in the relationship between cannabis and cognition may vary based on these ratios. Indeed, animal work suggests that long-term cognitive deficits due to cannabis were reversed with treatment of cannabidiol [65]. In addition, human work has suggested that higher cannabidiol strains decreased cannabis-related craving post-intoxication [66]. Therefore, it is possible that adolescents are only more vulnerable to increased craving and drug-related learning at higher potencies of THC [39, 56]. Similarly, most cannabis research in humans focuses solely on the effects of smoking cannabis; however, oral ingestion of cannabinoids through the use of edibles (e.g., baked goods, candies, drinks) is highly prevalent among both adolescent and adult cannabis users [67, 68]. Importantly, edible cannabis products often have higher concentrations of THC and the metabolic process in the digestive tract leads to higher levels of pharmacologically active THC metabolites in the body than when smoked [69]. Research is needed to elucidate whether age-related effects of cannabis on cognition differ depending on the route of administration.

Furthermore, research on the effects of cannabis in adolescence should differentiate between developmental periods within adolescence. By treating adolescence unitarily, current research may be missing time windows when risk or resilience patterns shift within the adolescence period. For instance, evidence from alcohol research suggests that the effects of age on social behavior may be most pronounced early adolescent-exposed rats, compared to late adolescent-exposed rats [42]. Future rodent and human work would benefit from exploring age-related effects on the relationship between cannabis and cognition through non-linear data modeling strategies. This would allow for insight into whether risk and resilience patterns vary between early, mid, and late adolescence.

Given the methodological constraints and ethical considerations of human studies, especially for adolescents, the value of rodent studies is evident. However, although rodent studies allow us to assess dose–response effects of different cannabinoids and the effect of abstinence on brain and cognition in a way that is not possible in humans, cannabis use-related addictive behaviors remain difficult to study. That is, previous animal studies suggest that rodents do not acquire self-administration behavior with THC [70], despite some evidence suggesting that they do find THC rewarding in other paradigms (e.g., conditioned place preference) [71]. Successful self-administration of synthetic cannabinoid WIN 55,212-2 in Long Evans rats [72] and THC in squirrel monkeys [73] do provide possibilities for future research. However, the challenge of self-administration of THC in rodents further highlights the difficulties of translating findings from animal models to humans given the potential differences in the rewarding and aversive capacity of cannabinoids across species.

While the current review addressed novel questions in both humans and rodents, there are several notable limitations. First, given that some of our studies have 3-way interactions, our null findings may have been due to low power. Secondly, we only had a small sample of human studies (*n* = 6). Therefore, our conclusions should be interpreted cautiously, particularly for human work. Lastly, we only included published studies. Due to publication bias, significant age-related results may be over-represented in this review. Despite these limitations, we believe that our review used a novel approach and question to address whether public health risk for cannabis in terms of cognition is different for adolescents and adults. This review, along with future work, can help inform prevention work such as whether resources should be allocated toward delaying cannabis use if adolescents are indeed at greater risk.

## Concluding remarks

While this systematic review does not offer a conclusive answer to the question of whether age changes the relationship between cannabis and cognition, the novel review question, along with the inclusion of both human and rodent work, has allowed for the formation of important hypotheses to be addressed in future work. First, in humans, general executive functioning seems to be more impaired in adolescent, frequent cannabis users compared to adult, frequent cannabis users. Second, in humans, age-effects may be most prominent among very heavy and dependent users, which may suggest CUD-specific effects. Third, in humans, craving and inhibitory control may not decrease as much after cannabis intoxication in adolescents compared to adults. Lastly, in rodents, the age-effects of cannabis on learning appear to be reversible if followed by sustained abstinence. If these hypotheses prove correct, it could lead to important developments in targeted prevention strategies.

## Electronic supplementary material

Below is the link to the electronic supplementary material.

Supplementary material 1 (DOCX 16 KB)
