# Supplementary material for: Age-related differences in the impact of cannabis use on the brain and cognition: a systematic review
Source: Eur Arch Psychiatry Clin Neurosci. 2019 Jan 24;269(1):37–58. doi: 10.1007/s00406-019-00981-7 (PMC6394430; doi:10.1007/s00406-019-00981-7)
Supplement: Supplementary file 1 — Supplementary material 1 (DOCX 16 KB) [file 406_2019_981_MOESM1_ESM.docx]

**Appendix S1 Literature search**

Our literature search used Medline from the National Library of Medicine (United States of America), PsycInfo from the American Psychological Association, and Cochrane Library from John Wiley & Sons. The initial hits were downloaded from the databases (see Table 1, 2, & 3 for search strategy). We also conducted an independent search in google scholar, not using our search syntax, to identify any other articles that may have been missed with our syntax. This included screening abstracts and tracking citations in previous cannabis reviews and meta-analyses. We also looked at whether there were any papers that met our criteria within the reference sections of our included studies.

Medline (1,067 hits), PsycINFO (813 hits), and Cochrane Library (244 hits) databases generated a total of 2124 hits, before deduplication. After the initial download, the results were uploaded to Zotero reference manager. Then, results were combined and de-duplicated using a filter that displayed the creator, year, issue, pages, and volume of each article and then, using the merge function if necessary. Following this, results were uploaded to Rayyaan for blinded review. All articles were reviewed by author, CG, and 1/3 of the articles were randomly reviewed by author, ES. Consensus for discrepancies was reached by all authors (CG, ES, LK, & JC). Duplicates that were missed in Zotero were resolved in Rayyaan. The total number of references after deduplication was 1,482, including the addition of 1 hit identified through a separate search on google scholar. Results from the review were downloaded to excel (see *Fig 1.* for entire screening process).

Table 1 Search syntax and results from Medline database, accessed on July 19^th^, 2018

| **Medline**  *Ovid MEDLINE(R) and Epub Ahead of Print, In-Process & Other Non-Indexed Citations, and Daily 1946 to July 19, 2018*    **#1 Marijuana**  cannabis/ OR marijuana abuse/ OR marijuana smoking/ OR dronabinol/ OR cannabinoids/ OR (cannabi* OR marijuana OR marihuana OR THC OR tetrahydrocannabi* OR dronabinol).ti,ab,kf.    **#2 Cognition**  cognitive dysfunction/ OR cognition/ OR neuropsychology/ OR decision making/ OR memory/ OR memory, long-term/ OR memory, short-term/ OR spatial memory/ OR spatial learning/ OR neurocognition/ OR (cognit* OR brain* OR memory OR executive function* OR intellectual* function* OR learning OR conditioning OR aversion OR processing speed OR locomotor OR craving OR neuro*).ti,ab,kf.  **#3 Adults and adolescents**  adolescent/ OR young adult/ OR adult/ OR (adolesc* OR adult*).ti,ab,kf.    **#4 Study type**  longitudinal studies/ OR cross-sectional studies/ OR age factors/ OR (longitud* OR cross-sectional* OR group difference* OR vehicle OR age-related difference* OR age difference* OR (adolesc* ADJ3 adult*) OR follow up OR followup).ti,ab,kf.  **1 AND 2 AND 3 AND 4 1,067 results** |
| --- |

Table 2 Search syntax and results from PsychInfo database, accessed on July 19^th^, 2018

| **PsycINFO**  ***Ovid, 1806 to July 19th, 2018***  **#1 Marijuana**  cannabis/ OR marijuana/ OR marijuana usage/ OR tetrahydrocannabinol/ OR Cannabinoids/ OR (cannabi* OR marijuana OR marihuana OR THC OR tetrahydrocannabi* OR  dronabinol).ti,ab,id.    **#2 Cognition**  cognitive impairment/ OR cognitive ability/ OR neuropsychology/ OR cognitive assessment/ OR decision making/ OR memory/ OR long term memory/ OR short term memory/ OR spatial memory/ OR neurocognition/ OR (cognit* OR brain* OR memory OR executive function* OR intellectual* function* OR learning OR conditioning OR aversion OR processing speed OR locomotor OR craving OR neuro*).ti,ab,id.    **#3 Adults and adolescents**  (adolescence 13 17 yrs OR young adulthood 18 29 yrs OR adulthood OR thirties).ag. OR (adolesc* OR adult*).ti,ab,id.    **#4 Study type**  (longitudinal study OR followup study).md. OR longitudinal studies/ OR age differences/ OR group differences/ OR (longitud* OR cross-sectional* OR group difference* OR vehicle OR age-related difference* OR age difference* OR (adolesc* ADJ3 adult*) OR follow up OR followup).ti,ab,id.  **1 AND 2 AND 3 AND 4 813 results** |
| --- |

Table 3 Search syntax and results from Cochrane Library database, accessed on July 19^th^, 2018

| **Cochrane Library**  *Wiley Interscience*  **#1 Marijuana**  ("cannabi*" OR "marijuana" OR "marihuana" OR "THC" OR "tetrahydrocannabi*" OR "dronabinol"):ti,ab,kw    **#2 Cognition**  ("cognit*" OR "brain*" OR "memory" OR "executive function*" OR "intellectual* function*" OR "learning" OR "conditioning" OR "aversion" OR "processing speed" OR "locomotor" OR "craving" OR "neuro*"):ti,ab,kw    **#3 Adults and adolescents**  ("adolesc*" OR "adult*"):ti,ab,kw    **#4 Study type**  ("longitud*" OR "cross-sectional*" OR "group difference*" OR "vehicle" OR "age-related difference*" OR "age difference*" OR ("adolesc*" NEAR/2 "adult*") OR "follow up" OR "followup"):ti,ab,kw  **#1 AND #2 AND #3 AND #4 244 results (5 reviews, 187 trials, 1 econ. eval.)** |
| --- |
